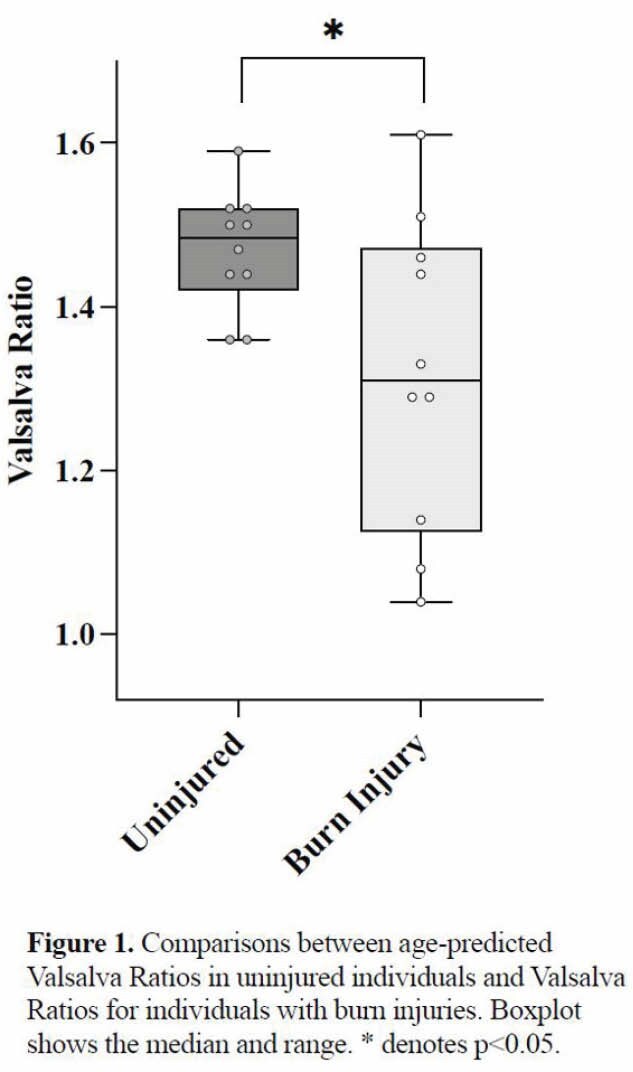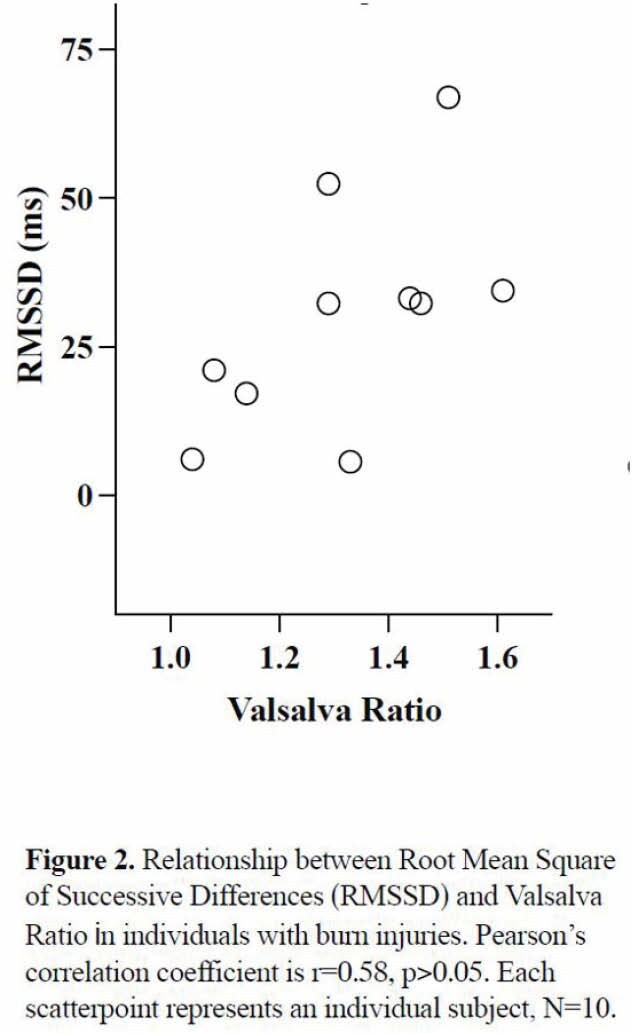# 871 Cardiac Parasympathetic Deficits in Individuals with Chronic Burn Injuries

**DOI:** 10.1093/jbcr/iraf019.402

**Published:** 2025-04-01

**Authors:** Huan Deng, Maria Sukhoplyasova, Kathryn Burns, Colleen Ryan, Jeffrey Schneider, J Andrew Taylor

**Affiliations:** Spaulding Rehabilitation Hospital; Spaulding Rehabilitation Hospital; Spaulding Rehabilitation Hospital; Shriners Children’s - Boston and Massachusetts General Hospital; Shriners Children’s - Boston and Massachusetts General Hospital; Spaulding Rehabilitation Hospital

## Abstract

**Introduction:**

Individuals with chronic burn injuries have a chronotropic incompetence in response to dynamic exercise. This may represent a deficit in cardiac sympathetic control due to decreased beta-adrenergic sensitivity. However, it is unknown if those with chronic burns also have cardiac autonomic deficits that extend to parasympathetic control. Therefore, this pilot work aimed to examine potential vagal deficits in those with chronic burns via standard tests of cardiac autonomic function.

**Methods:**

Adults with more than 10% total body surface area (TBSA) burned and 1-11 years after burn injury were recruited from the community. Supine subjects performed three Valsalva maneuvers which entailed forceful exhalation maintained at 40 mmHg with an open glottis for 15 seconds. This test characterizes changes in heart period and blood pressure consisting of four distinct phases. Valsalva ratio (VR) was derived from the maximum bradycardia and maximum tachycardia during the phase four blood pressure overshoot. This response represents the magnitude of baroreflex mediated cardiac vagal outflow in response to a blood pressure rise. Root Mean Square of Successive Differences (RMSSD) was derived from five minutes of resting heart period with uncontrolled breathing. RMSSD broadly reflects resting cardiac vagal modulation.

**Results:**

This study included 10 participants (7 males and 3 females), aged 21 to 59 years, with burns of 10% to 70% of TBSA (Mean ± SD: 34±15%) and with a post-injury duration of 3 to 11 years. VR was significantly lower than normative age-related values (1.32±0.19 vs 1.47±0.07, p< 0.05). Furthermore, the correlation between VR and RMSSD was close to reaching statistical significance (r=0.58, p=0.08).

**Conclusions:**

These preliminary data suggest that baroreflex mediated parasympathetic outflow is impaired in individuals with chronic burn injury. Moreover, lower baroreflex responses relate to lower resting cardiac vagal modulation, suggesting an overall parasympathetic deficit.

**Applicability of Research to Practice:**

These findings indicate that those with chronic burns have an autonomic profile suggestive of greater risk for long term cardiovascular morbidity and mortality.

**Funding for the Study:**

Spaulding Research Institute Research Accelerator program and NIDILRR #90DPBU0008.